# Validity of endoscopic ossiculoplasty immediately after its introduction for ossicular chain disruption

**DOI:** 10.1186/s12893-024-02445-9

**Published:** 2024-05-14

**Authors:** Atsushi Fukuda, Shinya Morita, Kimiko Hoshino, Keishi Fujiwara, Yuji Nakamaru, Akihiro Homma

**Affiliations:** Department of Otolaryngology-Head & Neck Surgery, Faculty of Medicine and Graduate School of Medicine, Hokkaido University, North 15, West 7, Kita-Ku, Hokkaido, Sapporo, 060-8638 Japan

**Keywords:** Congenital ossicular chain discontinuity, Ossiculoplasty, Transcanal endoscopic ear surgery, Traumatic ossicular chain dislocation

## Abstract

**Background:**

Transcanal endoscopic ear surgery (TEES) reportedly requires a long learning curve and may be associated with more complications and longer operative times than microscopic ear surgery (MES). In this study, we aimed to examine the usefulness and validity of TEES for ossicular chain disruption in the early stages of its introduction in our institution.

**Methods:**

TEES was performed on 11 ears (10 with congenital ossicular chain discontinuity and 1 with traumatic ossicular chain dislocation), and MES was performed with a retroauricular incision on 18 ears (6 with congenital ossicular chain discontinuity and 12 with traumatic ossicular chain dislocation) in a tertiary referral center. Postoperative hearing results, operative times, and postoperative hospital length of stay were retrospectively reviewed. The Mann–Whitney *U* test and Fisher’s exact test was performed to compare variables between the TEES and MES groups. Pre- and postoperative air- and bone-conduction thresholds and the air–bone gap of each group were compared using the Wilcoxon signed-rank test. The Mann–Whitney *U* test and Wilcoxon signed-rank was performed to compare the pre- and postoperative air–bone gaps between the diagnoses.

**Results:**

No significant differences in the postoperative air-conduction thresholds, bone-conduction thresholds, air–bone gaps, or incidence of air–bone gap ≤ 20 dB were observed between the TEES and MES groups. The air-conduction thresholds and air–bone gaps of the TEES group significantly improved postoperatively. The air-conduction thresholds and air–bone gaps of the MES group also significantly improved postoperatively. No significant difference was observed in the operative times between the groups (TEES group: median, 80 min; MES group: median, 85.5 min). The TEES group had a significantly shorter postoperative hospital stay (median, 2 days) than the MES group (median, 7.5 days).

**Conclusions:**

TEES was considered appropriate for the treatment of ossicular chain disruption, even immediately after its introduction at our institution. For expert microscopic ear surgeons, ossicular chain disruption may be considered a suitable indication for the introduction of TEES.

## Background

Ossicular chain disruption presents with conductive hearing loss, and congenital middle ear malformations and traumatic ossicular dislocations are among the contributing factors. Conductive hearing loss caused by congenital middle ear anomalies occurs in approximately 1 in every 11,000 to 15,000 cases [1]. Although congenital middle ear anomalies are diverse in appearance, the classification proposed by Teunissen and Cremers in 1993 based on a surgical perspective has been widely adopted and discussed [2]. In particular, ossiculoplasty is expected to improve hearing in patients with class III anomalies, which are defined as congenital malformations of the ossicular chain with a mobile stapes footplate including discontinuity of the ossicular chain and/or attic fixation [1, 2]. In contrast, injury to the ossicular chain has various causes. The main causes of ossicular injury are insertion of a foreign body into the external ear canal and trauma to the temporal, parietal, or occipital region, with or without fracture of the temporal bone [3]. Outcomes of immediate or delayed ossiculoplasty include satisfactory hearing even though late-stage cases are usually associated with adhesion and fibrosis [3].

Recently, the usefulness of endoscopic ear surgery has been reported, and it is rapidly becoming popular worldwide [4–7]. Transcanal endoscopic ear surgery (TEES), which uses a high-definition video system, has many advantages compared with microscopic ear surgery (MES), including a wider field of view, higher magnification of anatomical structures, and clear visualization of anatomical areas that would be blind spots when using a microscope [8]. TEES is also less invasive because it does not require a retroauricular incision, even if the external auditory canal is narrow and curved [9]. Several systematic reviews and meta-analyses of the postoperative outcomes of tympanoplasty for the repair of perforated tympanic membranes and stapes surgery with TEES have revealed postoperative tympanic membrane closure rates and hearing outcomes comparable to those of MES [5–7]. These results indicated that ossiculoplasty during TEES is reasonable. Although some studies on endoscopic ossiculoplasty have been reported [10, 11], the efficacy and safety of endoscopic ossiculoplasty have not yet been fully evaluated. TEES requires a long learning curve for many otologists because of its one-handed surgical technique and lack of depth perception [12]. Moreover, TEES is concerning because it might be associated with more complications and longer operative times than MES.

Although various otological surgeries have been performed using microscopes, TEES is considered a suitable treatment, especially for congenital ossicular chain discontinuity and traumatic ossicular chain dislocation, because these conditions are not usually associated with much inflammation and the procedure is mainly limited to the mesotympanum. We started TEES on a trial basis in 2018; in 2020, we introduced it as a regular treatment. Since then, TEES has been the first choice of treatment for all cases of ossicular chain disruption. During this study, we compared the postoperative results of TEES with those of conventional MES for ossicular chain disruption immediately after the introduction of TEES at our institution; furthermore, we examined the usefulness and appropriateness of TEES in the early stages of introduction. If this study clarifies the usefulness of TEES for ossicular chain disruption, it will expand the options for effective surgical procedures for ossicular chain disruption.

## Methods

This study was approved by the Institutional Review Board of Hokkaido University Hospital (Sapporo, Japan) for clinical research (IRB no. 022–0013), and it was conducted in accordance with the tenets of the Helsinki Declaration. We have obtained written informed consent from each participant or each participant's guardian.

### Aim

To compare TEES with conventional MES for ossicular chain disruption and to examine the usefulness and appropriateness of TEES in the early stages of introduction at our institution.

### Participants

We retrospectively reviewed the medical charts of patients with ears affected by congenital ossicular chain discontinuity or traumatic ossicular chain dislocation, who underwent primary ossiculoplasty during either TEES or MES at the Department of Otolaryngology, Hokkaido University Hospital, between May 2015 and January 2022. The only criterion for selecting TEES or MES was the timing of the surgery. Patients treated before February 2020 underwent conventional MES, whereas those treated after March 2020 underwent TEES. All TEESs and MESs were performed by two surgeons who had each been involved in over 300 cases and were proficient in MES but performed their first TEES during the study period. All patients had conductive or mixed hearing loss. Patients with a perilymphatic fistula or stapes fixation were excluded from the study. Patients with external ear anomalies or craniofacial malformations were also excluded. All patients underwent temporal bone computed tomography before surgery and were divided into the TEES group (those who underwent TEES) and MES group (those who underwent MES).

### Surgical procedures

All procedures were performed under general anesthesia. TEES was conducted using a transcanal approach with 0-degree or 30-degree angled rigid endoscopes with an outer diameter of 2.7 mm and a length of 180 mm (Karl Storz, Tuttlingen, Germany). The entire TEES procedure was performed using one hand. A circumferential endomeatal incision was first created in the middle of the bony ear canal. The tympanomeatal flap was elevated anteriorly, and ossicular chain abnormalities were evaluated endoscopically. The surgical ossiculoplasty procedures were determined based on the surgical findings. Ossiculoplasty was performed using autologous auricular cartilage and/or hydroxyapatite prostheses, and fibrin glue was used to stabilize the ossicular chain. The surgery was concluded by packing the bony ear canal with an absorbable gelatin sponge. Note that the use of bone cement or titanium prosthesis for ossiculoplasty is not covered by medical insurance in Japan. Also, although incus interposition is generally considered a good method for ossiculoplasty, it has not been adopted because it requires an additional atticotomy to remove the incus.

MES was performed using standard procedures via a transcanal approach with a postauricular incision. A postauricular incision is required for most Japanese patients because of their narrow and curved external auditory canals [9]. A tympanomeatal flap was created, the posterosuperior bony wall of the external auditory canal was drilled out as necessary, and ossicular chain abnormalities were evaluated using a microscope. The surgical steps of the ossicular chain evaluation and reconstruction were similar to those of TEES. The surgery was concluded by packing the bony ear canal with an absorbable gelatin sponge and ointment gauze.

### Clinical parameters

Data on patient characteristics (age and sex), diagnosis (congenital or traumatic), surgical procedures (TEES or MES), preoperative and postoperative hearing, intraoperative ossicular chain findings, ossiculoplasty type, operative times, complications, and postoperative hospital stay were reviewed. For cases of congenital ossicular discontinuity, the Teunissen and Cremers classification system [2] was used to define ossicular chain anomalies. The International Otology Outcome Group (IOOG) SAMEO-ATO scheme [13] was used to categorize ossicular chain reconstruction. The ossiculoplasty categories were defined by the furthest points of contact of the graft or prosthesis between these anatomical structures: m, malleus handle; t, tympanic membrane; I, incus; s, superstructure of stapes; and f, footplate of stapes (e.g., Ost indicates that the graft or prosthesis is placed between the superstructure of the stapes and tympanic membrane). The operative time was measured from the first incision until the completion of external auditory canal packing.

### Hearing outcomes

Hearing outcomes were assessed according to the Committee on Hearing and Equilibrium guidelines of the American Academy of Otolaryngology-Head and Neck Surgery (AAO-HNS) [14]. Pure-tone air-conduction and bone-conduction thresholds were obtained with thresholds at 0.5, 1, 2, and 3 kHz, which were used to calculate the pure-tone average air-conduction (AC) and bone-conduction (BC) thresholds and the pure-tone average air–bone gap (ABG). When the 3-kHz threshold was not tested, the mean thresholds at 2 and 4 kHz were used instead, and four-frequency (0.5, 1, 2, and 3 kHz) AC and BC thresholds and the ABG were calculated. Audiograms at ≥ 5 months after surgery were used to determine postoperative hearing.

### Statistical analyses

The Mann–Whitney *U* test was performed to compare age, operative time, postoperative hospital stay, preoperative and postoperative AC and BC thresholds, and the ABG between the TEES and MES groups. Fisher’s exact test was performed to compare the sex, diagnosis, ossiculoplasty type, and number of ears with a postoperative ABG of ≤ 20 dB between the TEES and MES groups. The preoperative and postoperative AC and BC thresholds and ABG of each group were compared using the Wilcoxon signed-rank test. The Mann–Whitney *U* test was performed to compare the preoperative and postoperative ABGs between the diagnoses. The Wilcoxon signed-rank test was also performed to compare the preoperative and postoperative ABGs for each diagnosis. Statistical significance was set at *p* < 0.05. All statistical analyses were performed using JMP Pro 16 software (SAS Institute, Inc., Cary, NC, USA).

## Results

A total of 29 ears (11 ears of 9 patients in the TEES group and 18 ears of 17 patients in the MES group) were enrolled in this study (Table 1). The median age of the TEES group was 18 (range, 6–65) years, whereas that of the MES group was 32.5 (range, 8–66) years. Regarding congenital ossicular chain discontinuity, the mean and median ages at surgery were 25.3 and 16 (range, 6–65) years in the TEES group, and 20.3 and 11.5 (range, 8–66) years in the MES group. The TEES group comprised four ears of four male patients and seven ears of five female patients. The MES group comprised 12 ears of 12 male patients and 6 ears of 5 female patients. The age and sex did not differ significantly between the groups (*p* = 0.38 and *p* = 0.14, respectively). Ten ears with congenital ossicular chain discontinuity and one with traumatic ossicular chain dislocation were treated with TEES. Six ears with congenital ossicular chain discontinuity and 12 with traumatic ossicular chain dislocation were treated with MES. The TEES group had a significantly higher proportion of ears with congenital ossicular chain discontinuities than the MES group (*p* = 0.0057). All congenital malformations were classified as class III according to the classification proposed by Teunissen and Cremers [2]. None of the patients had surgical findings that excluded them from ossiculoplasty.

**Table 1:** Comparisons of ears that underwent TEES and those that underwent MES

	TEES	MES	*p* value
Age, years, median (range)	18 (6–65)	32.5 (8–66)	0.38^a^
Sex, number of ears			0.14^b^
Male	4 (36.4%)	12 (66.7%)	
Female	7 (63.6%)	6 (33.3%)	
Diagnosis, number of ears			0.0057^b^
Congenital	10 (90.9%)	6 (33.3%)	
Traumatic	1 (9.1%)	12 (66.7%)	
Preoperative AC, dB HL, mean ± SD	54.9 ± 21.6	54.5 ± 16.6	0.96^a^
Preoperative BC, dB HL, mean ± SD	18.8 ± 16.9	20.0 ± 15.5	0.62^a^
Preoperative ABG, dB HL, mean ± SD	36.2 ± 13.8	34.5 ± 14.6	0.72^a^

*ABG* Air–bone gap, *AC* Air-conduction threshold, *BC* Bone-conduction threshold, *MES* Microscopic ear surgery, *SD* standard deviation, *TEES* Transcanal endoscopic ear surgery

^a^Differences were analyzed using the Mann–Whitney *U* test

^b^Differences were analyzed using Fisher’s exact test

Table 1 shows the preoperative hearing of the patients. The preoperative mean AC thresholds of the TEES and MES groups were 54.9 ± 21.6 dB HL and 54.5 ± 16.6 dB HL, respectively. The preoperative mean BC thresholds of the TEES and MES groups were 18.8 ± 16.9 dB HL and 20.0 ± 15.5 dB HL, respectively. The preoperative mean ABGs of the TEES and MES groups were 36.2 ± 13.8 dB HL and 34.5 ± 14.6 dB HL, respectively. The preoperative AC threshold, BC threshold, and ABG did not differ significantly (*p* = 0.96, *p* = 0.62, and *p* = 0.72, respectively) between the TEES and MES groups.

Table 2 shows a comparison of ossiculoplasty types, postoperative hearing results, operative times, and postoperative hospital stays of the TEES and MES groups. All cases of middle ear malformation had only ossicular chain discontinuity findings, and none were complicated by attic fixation. Ossicular chain repair was categorized based on the IOOG SAMEO-ATO scheme [13]. No significant differences in the ossiculoplasty types (*p* = 0.32) were observed (TEES group: Ost, 7 ears; Osi, 1; Oft, 2; and Ofm, 1; MES group: Ost, 15 ears; Osi, 1; Oft, 0; and Ofm, 2).

**Table 2:** Comparison of intraoperative and postoperative results of TEES and MES

	TEES	MES	*p* value
Ossiculoplasty^c^, number of ears			0.32^a^
Ost	7 (63.6%)	15 (83.3%)	
Osi	1 (9.1%)	1 (5.6%)	
Oft	2 (18.2%)	0 (0%)	
Ofm	1 (9.1%)	2 (11.1%)	
Postoperative AC, dB HL, mean ± SD	28.5 ± 22.5	30.3 ± 14.3	0.27^b^
Postoperative BC, dB HL, mean ± SD	15.4 ± 15.5	18.2 ± 15.6	0.54^b^
Postoperative ABG, dB HL, mean ± SD	13.1 ± 10.6	12.2 ± 6.8	0.82^b^
Postoperative ABG ≤ 20 dB, number of ears	9 (81.8%)	17 (94.4%)	0.54^a^
Congenital	8 (80.0%)	5 (83.3%)	
Traumatic	1 (100%)	12 (100%)	
Operative time, min, median (range)	80 (38–120)	85.5 (63–137)	0.31^b^
Postoperative hospital stay, days, median (range)	2 (1–7)	7.5 (2–10)	0.0003^b^

*m* malleus handle,*t* tympanic membrane, *I* Incus, *s* superstructure of stapes and *f* footplate of stapes. *ABG* Air–bone gap, *AC* Air-conduction threshold, *BC* Bone-conduction threshold, *MES* Microscopic ear surgery, *SD* Standard deviation, *TEES* Transcanal endoscopic ear surgery

^a^Differences were analyzed using the Mann–Whitney *U* test

^b^Differences were analyzed using Fisher’s exact test

^c^Ossicular chain reconstruction was categorized according to The International Otology Outcome Group SAMEO-ATO scheme [13]

**Fig. 1:**
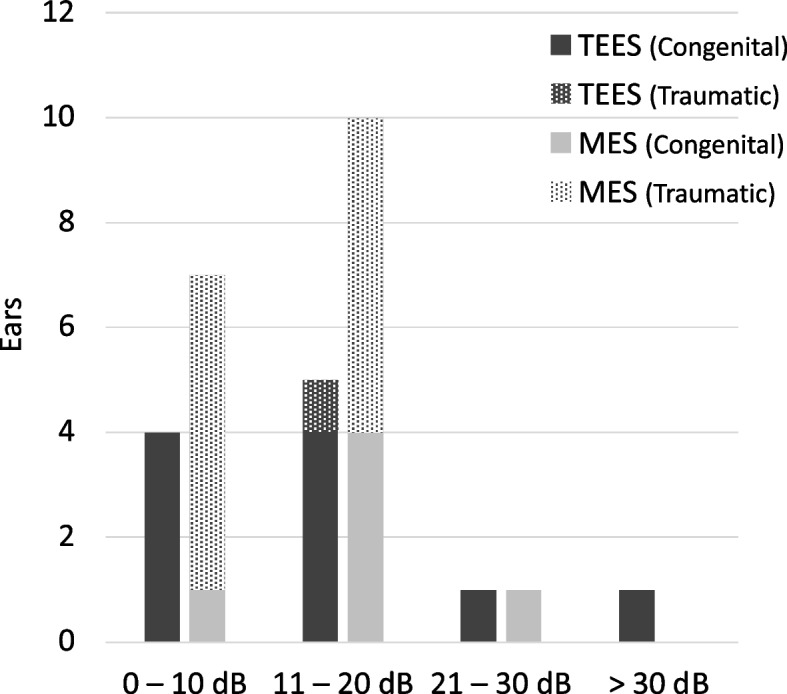
Postoperative air–bone gap (ABG) closure after ossiculoplasty for ossicular chain disruption. Postoperative ABG closure of ≤ 20 dB was achieved in 9 (81.8%) ears in the transcanal endoscopic ear surgery (TEES) group and 17 (94.4%) ears in the microscopic ear surgery (MES) group

**Fig. 2:**
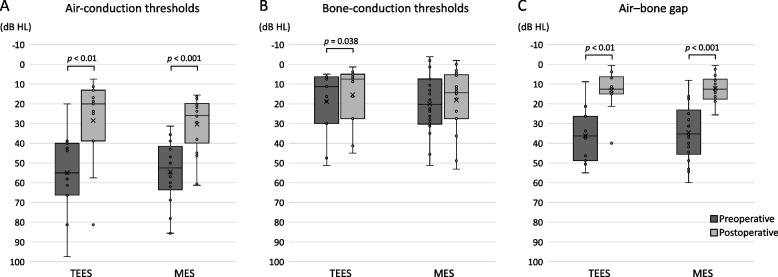
Comparisons of the preoperative hearing results and hearing results after TEES and MES. In the TEES group, the postoperative air-conduction (AC) threshold (**a**), bone-conduction threshold (**b**), and air–bone gap (ABG) (c) showed significant improvement compared with the preoperative values. In the MES group, the postoperative AC threshold (**a**) and ABG (**c**) showed significant improvement compared with the preoperative values. Differences were analyzed using the Wilcoxon signed-rank test. MES, microscopic ear surgery; TEES, transcanal endoscopic ear surgery

The median time to postoperative audiometry was 13 (range, 5–20) months. The postoperative mean AC thresholds of the TEES and MES groups were 28.5 ± 22.5 dB HL and 30.3 ± 14.3 dB HL, respectively. The postoperative mean BC thresholds of the TEES and MES groups were 15.4 ± 15.5 dB HL and 18.2 ± 15.6 dB HL, respectively. The postoperative mean ABGs of the TEES and MES groups were 13.1 ± 10.6 dB HL and 12.2 ± 6.8 dB HL, respectively. Figure 1 shows the postoperative ABG closure according to the AAO-HNS guidelines. A postoperative ABG of ≤ 20 dB was observed in 9 (81.8%) ears in the TEES group and 17 (94.4%) in the MES group. In the TEES group, a postoperative ABG of ≤ 20 dB was achieved in eight (80.0%) ears for congenital ossicular chain discontinuity and in one (100%) ear for traumatic ossicular chain dislocation. Whereas in the MES group, postoperative ABG ≤ 20 dB was achieved in five (83.3%) ears for congenital ossicular chain discontinuity and in 12 (100%) ears for traumatic ossicular chain dislocation. Thus, in both groups, the rates of achieving postoperative ABG ≤ 20 dB were comparable for congenital ossicular chain discontinuity and traumatic ossicular chain dislocation, respectively. No significant differences in the postoperative AC thresholds, BC thresholds, ABGs, or incidence of ABG ≤ 20 dB (*p* = 0.27, *p* = 0.54, *p* = 0.82, and *p* = 0.31, respectively) were observed between the groups. Figure 2 compares the preoperative and postoperative AC thresholds, BC thresholds, and ABGs of the TEES and MES groups. In the TEES group, the AC threshold, BC threshold, and ABG significantly improved postoperatively (*p* < 0.01, *p* = 0.038, and *p* < 0.01, respectively). In the MES group, the AC threshold and ABG significantly improved postoperatively (*p* < 0.001 and *p* < 0.001, respectively).

**Fig. 3:**
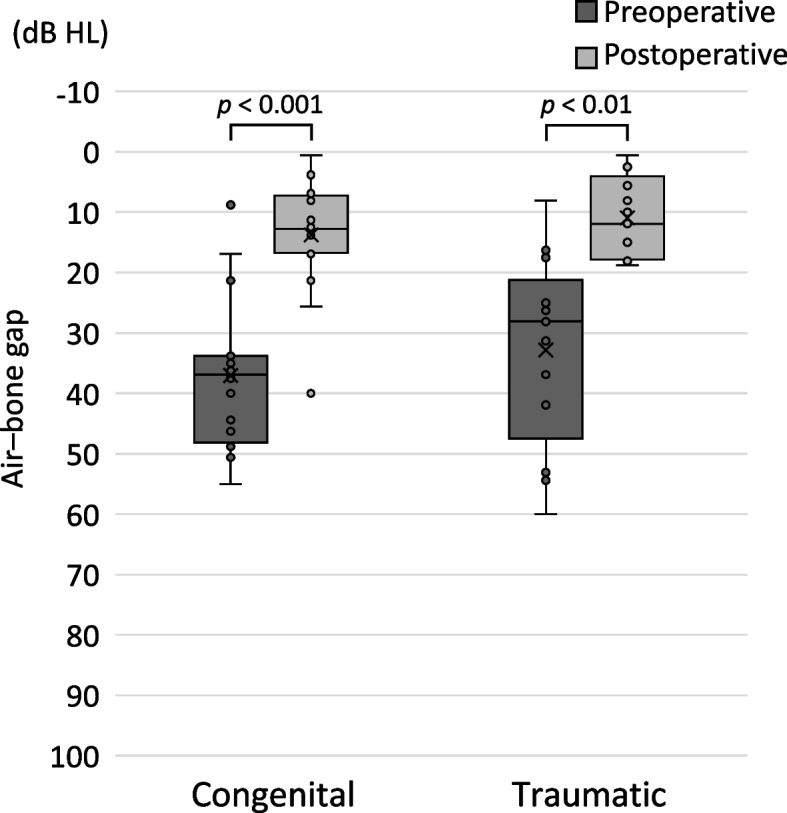
Pre- and postoperative ABGs associated with congenital ossicular chain discontinuity and traumatic ossicular chain dislocation. The congenital ossicular chain discontinuity and traumatic ossicular chain dislocation groups did not differ in terms of the preoperative and postoperative ABGs. The ABGs associated with congenital ossicular chain discontinuity and traumatic ossicular chain dislocation improved significantly with the procedure. ABG, air–bone gap

Figure 3 shows a comparison of preoperative and postoperative ABGs according to the diagnosis. The preoperative and postoperative mean ABGs were 37.0 ± 12.7 dB HL and 13.8 ± 9.4 dB HL, respectively, for congenital ossicular chain discontinuity and 32.8 ± 15.8 dB and 11.0 ± 6.7 dB HL, respectively, for traumatic ossicular chain dislocation. The preoperative and postoperative ABGs did not differ significantly between congenital ossicular chain discontinuity and traumatic ossicular chain dislocation (*p* = 0.35 and *p* = 0.71, respectively). The postoperative ABGs associated with congenital ossicular chain discontinuity and traumatic ossicular chain dislocation were significantly improved compared with the preoperative ABGs (*p* < 0.001 and *p* < 0.01, respectively).

The operative times of the TEES group (median, 80 min; range, 38–120 min) and MES group (median, 85.5 min; range, 63–137 min) were not significantly different (*p* = 0.31). The intraoperative and postoperative complications included chorda tympani severance in one ear in the MES group. No cases of postoperative BC threshold deterioration > 10 dB in either group were observed. No cases of postoperative facial nerve paralysis, sensorineural hearing loss, or vertigo were observed. The postoperative hospital stay of the TEES group (median, 2 days; range, 1–7 days) was significantly shorter than that of the MES group (median, 7.5 days; range, 2–10 days) (*p* = 0.0003).

## Discussion

During this study, we compared the postoperative hearing results, operative times, complications, and postoperative hospital stays associated with TEES and conventional MES for congenital ossicular chain discontinuity and traumatic ossicular chain dislocation to investigate the efficacy of endoscopic ossiculoplasty in the early stages of introduction. Previously reported postoperative hearing success rates with an ABG of ≤ 20 dB achieved with MES for class III middle ear anomaly based on the Teunissen and Cremers classification ranged from 74 to 90% [2, 15]. A class III middle ear anomaly with this classification indicated a congenital malformation of the lateral ossicular chain, including discontinuity of the ossicular chain and/or attic fixation with a mobile stapes footplate. Using the same criteria, Ito et al. [16] reported that the postoperative hearing success rates of TEES and MES for class III congenital ossicular anomalies were 85.7% and 100%, respectively, with no significant differences. Chung et al. [17] also reported equivalent hearing outcomes after TEES and MES for class III congenital ossicular anomalies, with a postoperative ABG within 10 dB achieved for 75.0% and 71.4% of cases, respectively. In contrast, Kim et al. [18] performed TEES for traumatic ossicular chain dislocation and reported that a postoperative ABG within 20 dB was achieved for 100% of cases. In this study, 81.8% of patients in the TEES group and 88.9% of those in the MES group had a postoperative ABG within 20 dB, with no significant differences in postoperative hearing outcomes between the groups. These results were comparable to those reported previously, even though simple comparisons cannot be made because patients with a class III congenital ossicular anomaly with no discontinuity but fixation of the malleus and/or incus were not included in this study. The present data showed TEES to be appropriate for congenital ossicular chain discontinuity, and while evidence for traumatic cases was insufficient, we have no reason to believe that it would not work in such cases as well, because hearing results did not differ between traumatic and congenital cases and the treatment of one patient with traumatic ossicular chain dislocation in the TEES group went well. Therefore, the hearing results of ossiculoplasty during TEES for ossicular disruption may be comparable to those of ossiculoplasty during conventional MES and considered satisfactory, regardless of whether the cause is a congenital malformation of the middle ear or trauma.

Ito et al. [16] reported that the operative times of MES and TEES for congenital middle ear anomalies were comparable. However, Chung et al. [17] reported a significantly shorter operative time for TEES. During this study, the operative time required to perform ossiculoplasty for ossicular chain disruption did not differ significantly between the TEES and MES groups. In their large study of intraoperative and postoperative complications associated with various endoscopic ear surgeries (tympanoplasty, myringoplasty, stapedoplasty, canaloplasty, ossiculoplasty, and exploratory tympanotomy), Marchioni et al. [19] confirmed the safety and very low complication rates of the endoscopic technique. During this study, chorda tympani severance was observed in only one ear in the MES group, and no serious complications, such as facial paralysis, occurred. In the present study, postoperative BC threshold improved in the TEES group. However, it is not theoretical that BC threshold will improve after ossiculoplasty in cases of ossicular chain disruption, whether congenital or traumatic. Therefore, this result was clinically meaningful in that BC threshold did not significantly worsen postoperatively. This means that despite the longer middle ear maneuver time in TEES compared to MES, there was no damage to the inner ear that would lead to a deterioration in BC threshold. TEES is a one-handed procedure; therefore, the surgical maneuvers are more time-consuming and difficult than those of MES. However, ossiculoplasty for ossicular chain disruption performed via TEES is unlikely to require more time or pose higher risks than that performed via MES with a postauricular incision.

During this study, the TEES group had a significantly shorter postoperative hospital stay. Short-stay surgery is not as prevalent in Japan as it is in the West because of differences in medical fees, healthcare insurance, and other systems; therefore, postoperative hospital stays for otologic surgery under general anesthesia are usually approximately 1 week in Japan. At our institution, ointment gauze was packed into the ear canal at the end of MES, and it was removed approximately 5–7 days later; a large percentage of patients preferred to be discharged after the gauze was removed. In contrast, TEES does not require packing of the ear canal with gauze. Furthermore, TEES has been reported to cause less postoperative pain than MES with a retroauricular incision [20]; thus, the reassurance of a smaller wound and less pain might have contributed to the shorter time to discharge. TEES may contribute to lower healthcare costs because it is associated with shorter hospital stays and smaller wounds, which are associated with a lower incidence of problems immediately after discharge.

In this study, TEES was performed after March 2020 and was the first series in which surgeons used endoscopes. Therefore, the surgeons were experts in the practice of the MES group, but were considered novices in the practice of TEES. However, they achieved postoperative outcomes comparable to those of conventional MES. With more experience and further proficiency with TEES, it is believed that a more desirable surgical outcome than MES can be fully expected in the future.

This study had some limitations. First, its retrospective design might have affected our results. Second, the causes of ossicular chain disruption experienced by patients in the TEES and MES groups were subject to bias; however, no significant differences were found in the preoperative and postoperative ABGs between congenital ossicular chain discontinuity and traumatic ossicular chain dislocation. Further prospective studies are required to improve our understanding of the efficacy and validity of TEES.

## Conclusions

In conclusion, the hearing outcomes after TEES and MES for ossicular disruption were comparable even immediately after the introduction of TEES at our institution, and they were comparable to those of previous studies performed at other institutions. The operative times were also comparable between the groups. The postoperative hospital stay of the TEES group was significantly shorter than that of the MES group, suggesting that TEES is less invasive and burdensome than MES. For expert microscopic ear surgeons, ossicular chain disruption may be considered a suitable indication for the introduction of TEES. This study indicates that TEES is useful for ossicular chain disruption and is an effective surgical option for ossicular chain disruption.

## Data Availability

The data that support the findings of this study are not publicly available but are available from the corresponding author upon reasonable request.

## Abbreviations

AAO-HNS: American Academy of Otolaryngology-Head and Neck Surgery
ABG: Air–bone gap
AC: Air conduction
BC: Bone conduction
IOOG: International otology outcome group
MES: Microscopic ear surgery
TEES: Transcanal endoscopic ear surgery
